# Urethral Triplication Without Bladder Duplication: Endourologic Diagnosis and Management

**DOI:** 10.1089/cren.2018.0019

**Published:** 2018-04-01

**Authors:** Alberto Parente, Ruben Ortiz, Laura Burgos, Jose Maria Angulo

**Affiliations:** Department of Pediatric Urology, Gregorio Marañon University Hospital, Madrid, Spain.

**Keywords:** urethral, triplication, children, urethral duplication

## Abstract

Urethral triplication is a rare congenital anomaly of the lower urinary system, with <15 cases reported so far. We present a 24-month-old boy with accessory hypoplastic urethra ending in glans. At the beginning of toilet training, urine output was observed along the rectum. Rigid cystoscopy shows a perineal urethra starting in the posterior urethra. Subsequently, flexible cystoscopy showed entry of epispadic urethra in the bladder immediately superior to the bladder neck. It was running parallel to primary urethra. Then, we observed two most frequent types of urethral duplication in the sagittal plane in a single patient.

## Introduction

Urethral duplication is a rare congenital anomaly of the lower urinary system. Urethral triplication is extremely rare,^1,2^ with <15 cases reported until now.^2^ In general, it is associated with other urinary malformations such as bladder duplication, vesicoureteral reflux, or urethrodeferential reflux.^3,4^

Unlike urethral duplication, ureteral triplication tends to be symptomatic. These symptoms include incontinence, urinary tract infection (UTI), and triple urinary stream. The presentation depends on the anatomy of the anomaly. We report a new case of urethral triplication.

## Case Report

A 24-month-old boy was referred to our pediatric urology clinic with a history of bilateral vesicoureteral reflux. Right kidney had no function. Antibiotic prophylaxis was initiated and circumcision was performed in newborn age. After performing circumcision, we discovered the existence of an accessory hypoplastic urethra ending in the glans.

**FIG. 1.**
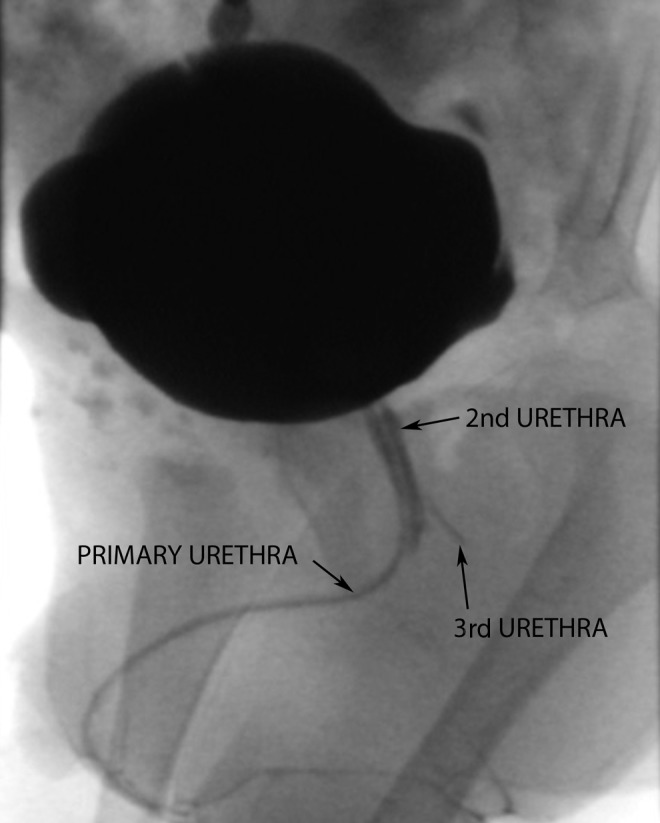
Voiding cystourethrogram, observing hypoplastic urethra running parallel to the main urethra (2nd urethra), and perineal urethra in Y (3rd urethra).

Despite antibiotic prophylaxis, the patient suffered recurrent UTIs. At the beginning of toilet training, urine output was observed through the rectum. Simultaneously with the urinary stream, minimal urine drops were seen through the epispadic urethra. No other physical abnormalities were observed. A new cystography was performed, confirming the previously known two urethras and, in addition, a third urethra was seen from posterior urethra to perineum in “Y” shape (Fig. 1).

**FIG. 2.**
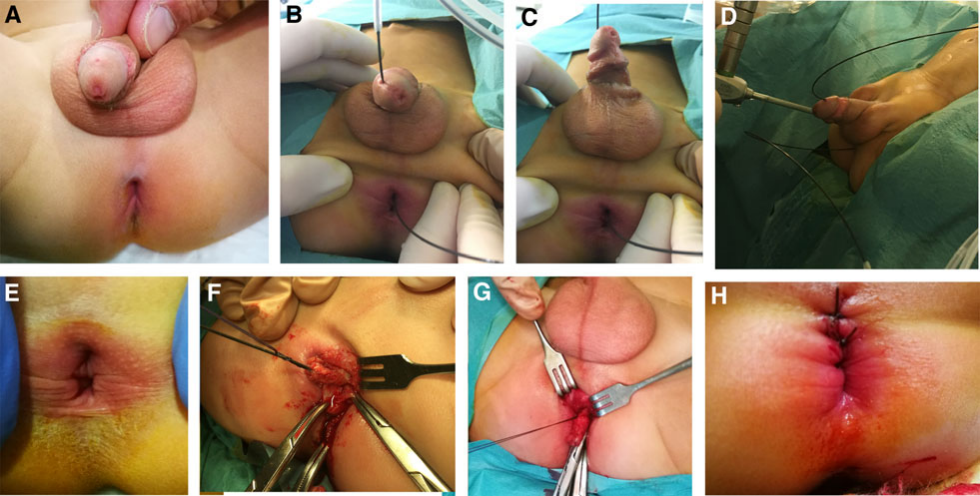
**(A–D)** The patient was found to have an epispadic urethra (2nd urethra) and perineal urethra (3rd urethra). **(E–H)** Surgical treatment was performed on the perineal urethra.

Further examination revealed that the primary glans meatus accepted a 10F urethral catheter, the epispadias pit on the dorsal glans meatus accepted a 4F catheter, and the perineal urethra accepted a 5F urethral catheter (Fig. 2).

**FIG. 3.**
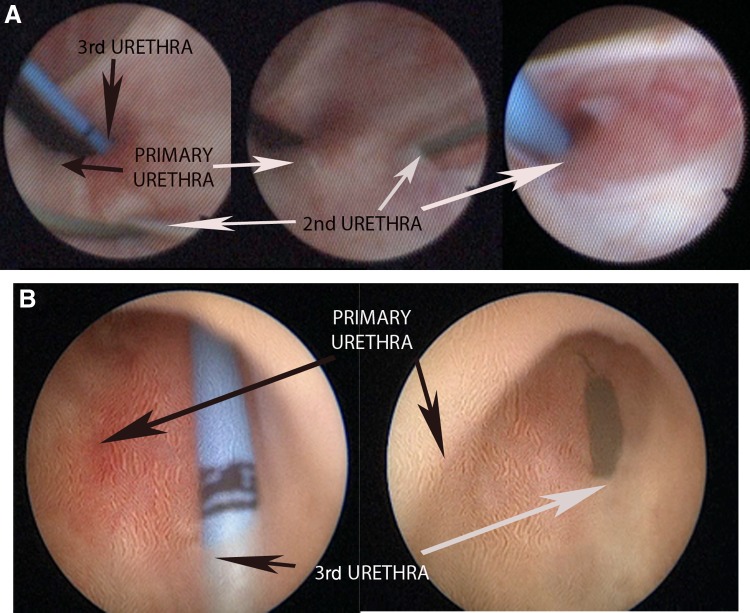
**(A)** Flexible cystoscopy (retrograde vision when rotating 180 grades the flexible ureteroscope) shows the entry of epispadic urethra into the bladder immediately superior to the bladder neck (2nd urethra). The main urethra and perineal urethra (3rd urethra) enter the bladder through the bladder neck. **(B)** Rigid cystoscopy shows a perineal urethra (3rd urethra) starting in the posterior urethra. Entrance of the perineal urethra in the primary urethra was coagulated under cystoscopic control.

Cystoscopy was performed and accessory urethras were identified. Rigid cystoscopy showed a perineal urethra starting in the posterior urethra. Subsequently, flexible cystoscopy with flexible microureteroscopy was performed, which showed that the epispadic urethra ran parallel to the primary urethra and enters the bladder immediately superior to the bladder neck (Fig. 3).

It was decided to perform surgical treatment of the perineal urethra to achieve urinary continence and avoid urinary infections. At the same time, we decided not to treat the epispatic urethra because of lack of symptoms.

Perineal approach was performed for dissection and removal of the perineal urethra (Fig. 2).

The entrance of the perineal urethra in the primary urethra was coagulated under cystoscopic control (Fig. 3) and an endoscopic treatment of vesicoureteral reflux was performed (Deflux^®^ injection).

The patient was discharged 24 hours postoperatively, remaining afebrile and with good pain control. Bladder catheter was kept in place through the main urethra for 7 days and it was removed in the outpatient clinic.

The patient had a single UTI a month later. After 1 year, he remains asymptomatic and dry without urinary leaks.

## Discussion

The urethra develops from the endodermal urogenital sinus, which is the anterior portion of the cloaca after it becomes separated from the posterior anorectal canal.

Urethral duplication is an infrequent urologic problem. This may occur with a sagittal or coronal pattern. The sagittal is more common than coronal pattern. In this case, the epispadic urethra was running above the primary urethra. In the coronal form, the duplicate urethras run parallel to each other. The most common sagittal pattern comprises an orthotopic principal urethral channel and an epispadiac hypoplasic urethra. It is also described that the accessory urethra emerges from the primary urethra and runs perineally in Y.^5^

Several theories have been proposed to explain the etiology and embryology of an accessory urethra, including urethral angle bifurcation by continuation of splitting of the urorectal septum, abnormal Müllerian duct termination, the occurrence of an ischemia process, growth failure of the urogenital sinus, and a lack of midline mesodermal fusion.^5^ Recently, van der Putte^3^ suggested the abnormal division of the urogenital sinus as the relative cause of urethral triplication. However, the pathogenesis of an accessory urethra is still unclear.

Although most patients with an accessory urethra will be asymptomatic, they could develop several complications, including outlet obstruction, a double or triple urinary stream, UTI, urinary incontinence, and renal insufficiency.^2,6^ In our case, we believe that the perineal urethra's location, ending next to the rectum, favored urinary infections. In addition, this urethra hinders urinary continence.

Radiographically, voiding cystourethrography is the election test. Oblique views and retrograde urethrography are mandatory to observe sagittal and coronal duplications.

However, in our reported patient, the urethral triplication was unnoticed during the neonatal period probably because of absence of clinical suspicion.

We detected urethras during surgery by insertion of a small caliber stent. In most of the published cases, this was impossible because part of accessory urethras were extremely hypoplasic. In our case, the patient was able to urinate through the three urethras.

The treatment of accessory urethras varies according to their anatomy and clinical manifestations.^6^ The principle is to reserve or rebuild a primary urethra that allows a satisfactory urinary stream. In asymptomatic patients, a good urinary stream would usually make surgical treatment unnecessary. In our child, the epispadiac urethra was asymptomatic and the esthetic defect was minimal.

In conclusion, we think that in our case we observed the two most frequent types of urethral duplication in the sagittal plane in a single patient. The absence of cases in the literature prevents creating a more specific classification and more standardized treatment guidelines.

## Conclusion

In this case, we observed the two most frequent types of urethral duplication in the sagittal plane in a single patient. Endourologic management could help complete the diagnosis and decrease the morbidity of corrective surgery.

## Authors' Contributions

All authors substantially participated in the conception, design, and execution of the study, data analysis and interpretation, and drafting and editing of the article.

## Abbreviation Used

UTI: urinary tract infection
